# On the role of allergen-specific IgG subclasses for blocking human basophil activation

**DOI:** 10.3389/fimmu.2022.892631

**Published:** 2022-10-06

**Authors:** Simon Zinkhan, Franziska Thoms, Gilles Augusto, Monique Vogel, Martin F. Bachmann

**Affiliations:** ^1^ Department of Immunology, University Clinic of Rheumatology and Immunology, Inselspital, University of Bern, Bern, Switzerland; ^2^ Department of BioMedical Research, University of Bern, Bern, Switzerland; ^3^ Saiba Animal Health, Pfäffikon, Switzerland; ^4^ Nuffield Department of Medicine, The Henry Wellcome Building for Molecular Physiology, The Jenner Institute, University of Oxford, Oxford, United Kingdom

**Keywords:** allergen-specific immunotherapy, IgG1, IgG4, FcγRIIb, allergen, basophils, basophil activation

## Abstract

Successful treatment of IgE mediated allergies by allergen-specific immunotherapy (AIT) usually correlates with the induction of allergen-specific IgG4. However, it is not clear whether IgG4 prevents the allergic reaction more efficiently than other IgG subclasses. Here we aimed to compare allergen-specific monoclonal IgG1 and IgG4 antibodies in their capacity to inhibit type I allergic reactions by engaging FcγRIIb. We found that IgG1, which is the dominant subclass induced by viruses, binds with a similar affinity to the FcγRIIb as IgG4 and is comparable at blocking human basophil activation from allergic patients; both by neutralizing the allergen as well as engaging the inhibitory receptor FcγRIIb. Hence, the IgG subclass plays a limited role for the protective efficacy of AIT even if IgG4 is considered the best correlate of protection, most likely simply because it is the dominant subclass induced by classical AITs.

## Introduction

The World Health Organization (WHO) identifies chronic respiratory diseases including allergies and chronic rhinosinusitis as one of the four major diseases affecting the world’s population (1). According to the ARIA initiative (Allergic Rhinitis and its Impact on Asthma), IgE-mediated inflammation of the nasal mucosa defines allergic rhinitis and causes the respective symptoms upon allergen exposure (2). Allergies are characterized by two phases: sensitization causing development of allergen-specific memory Th2 and B cells as well as the production of allergen-specific IgE at the early stage and effector functions associated with tissue inflammation and damage at later stages. Patients suffering from allergies usually treat their symptoms with antihistamines or corticosteroids or try to reduce allergen exposure by their avoidance and use of high-efficiency particulate air (HEPA) filters or temperature-controlled laminar airflows. However, for actual treatment of type I allergies, such as allergies against pollen, house dust mite, pet dander, food, or venom toxins, allergen-specific immunotherapy (AIT) represents the only disease modifying option addressing the cause of the illness (2–5). Commonly, allergens are administered subcutaneously or sublingually but also novel routes such as epicutaneous and intra-lymphatic have been established for AIT (6–12). AIT usually requires numerous allergen applications over 3-5 years (13, 14). The main disadvantages are the duration of the therapy and the risk of severe side effect such as anaphylactic reactions. However, successful AIT induces immune unresponsiveness to allergens and affects rhino-conjunctival symptoms and improves asthmatic conditions. AIT does not only mediate short-term but also long-term protection and is qualified as disease-modifying therapy leading overall reduced allergy disease severity, reduced less drug consumption and prevention of future allergen sensitization (2–5, 15–19). In addition to novel routes of allergen administration, clinical testing of AIT strives for safer and more efficient therapy conditions including use of allergens or allergoids formulated with stronger adjuvants (20–25) or use of peptides rather than full-length allergens (26–29). In addition, we have recently proposed that allergens displayed on virus-like particles may be a safe and efficacious alternative to standard AIT protocols (26, 30, 31).

The therapeutic mechanisms of AIT still remain a matter of debate. While some argue that induction of allergen-specific IgG antibodies is the key, as they can both neutralize the allergen and engage the inhibitory receptor FcγRIIb (32), others prefer the hypothesis that regulatory T cells are the masters to keep mast cells and basophils at bay and promote the production of IgG antibodies by altered cytokine secretion (33). Recently, it has been demonstrated in mice (30) and humans (34) that monoclonal antibodies against Fel d 1, the major cat allergen, can abrogate cat allergy, clearly indicating that anti-allergen IgG antibodies can reduce allergic symptoms (34). Furthermore, it is clear that successful immunotherapy correlates with induction of allergen-specific IgG4 or IgG4/IgE ratio (35). This has led to the general consensus in the field that induction of IgG4 is the major goal of specific immunotherapy. Others, however, have argued that dominant induction of IgG4 merely reflects the way AIT is performed, namely by multiple injections of small amounts of allergen formulated in Alum and does not reflect a superior efficacy of IgG4 at neutralizing allergens or engaging FcγRIIb (12, 36). Recently it been shown that the affinity of the IgG antibody is important. The direct neutralization of allergen was more efficient with high affinity antibodies although the inhibition of degranulation *via* FcγRIIb could be accomplished with both high and low affinity (37). Here we demonstrate that IgG4 binds to FcγRIIb with similar efficiency as IgG1 and inhibits basophil activation with equivalent efficacy both *via* the FcγRIIb as well as the allergen-neutralization pathway. Hence, IgG subclasses play a limited role in the efficacy of AIT.

## Material and methods

### Production of recombinant Fel d 1 protein

The expression, production, and purification of recombinant Fel d 1 dimer was performed as described elsewhere (38).

### Native human and recombinant IgG1 and IgG4

By using mammalian cell display (39), isolation and generation of 3 mAbs recognizing the non-overlapping epitopes A044, F127, and G078 on Fel d 1 were recently described (40). Native human IgG1 (Cat No 184886) and IgG4 (Cat No 183266) were purchased from Abcam (Cambridge, UK) and reconstituted as per the manufacturer’s instructions.

### Basophil activation test with blood from cat-allergic and non-allergic subjects

Whole blood samples were collected in EDTA tubes. Degranulation of basophils was determined using the FLOW CAST Basophil Activation Test (Bühlmann Laboratories, Schönenbuch, Switzerland) with some modifications. Briefly, red blood was lysed using home-made Ammonium-Chloride-Potassium (ACK) lysing buffer before resuspension of the cells into RPMI+/+ medium (RPMI containing 10ng/ml IL-3 and 5% human AB serum) containing 5µM of DARPin 53_79 for 1 hour at 37°C to strip IgE from the cells (41). Samples were washed 3 x times with PBS to remove dissociated IgE and DARPin proteins. Stripped cells were re-sensitized with different monoclonal IgE (F127, A044 and G078) at 100 nM in RPMI+/+ for 1 hour at 37°C. For each BAT assay there was a spontaneous basophil activation of around 1-4%. Cells were washed 3 x times with PBS before incubating with either Fel d 1 alone at a fixed concentration (concentration was previously determined for every donor by a Fel d 1 titration curve; the concentration leading to ~75% of peak basophil activation was chosen for each subject) or Fel d 1 in complexed with IgG1 or IgG4 (F127, A044, G078) in stimulation buffer following the producer’s instructions of the FLOW CAST protocol for 25 min at 37°C. Samples were then analyzed by flow cytometry for CCR3 and CD63 expression.

Samples from cat allergic subjects after red blood lysis and resuspension in RPMI+/+ medium were directly incubated with Fel d 1 alone or in complex with IgG1 or IgG4 without re-sensitization with IgE.

To investigate the role of FcγRIIb, cells from non-allergic subjects were incubated after sensitization with IgE with an anti-FcγRIIb DARPin which blocks IgG interaction with FcγRIIb (DARPin 11_11) (42). For control purposes after sensitization with IgE cells were incubated with 50 μl of anti-FcεRI (a highly specific monoclonal antibody against IgE receptor) and 50 μl of fMLP (an unspecific cell activator – the chemotactic peptide N-Formyl-methionyl-leucyl-phenylalanine).

### IgG1 and IgG4 kinetics by Bio-Layer Interferometry (BLI)

The analysis of binding kinetics of human native IgG1 and IgG4 as well as of recombinant anti-Fel d 1 IgG1 and IgG4 monoclonal antibodies (F127, A044 and G078) to FcγRIIb was analyzed by Bio-Layer Interferometry (BLI) using an Octet RED96e (Sartorius) instrument. Briefly, high precision Streptavidin (SAX, Sartorius, Fremont, CA, USA) biosensors were saturated with 2 μg/ml of human recombinant biotinylated FcγRIIb (CD32b) (Sino Biological, Cat No 10259_H27H-BI, Beijing, China) in kinetics assay buffer (PBS, 0.1% BSA, 0.02% Tween 20) for 10 min. Association was carried out in 300 s, with the IgG serially diluted from 100 to 6.3 nM in 1:2 steps. Finally, dissociation was also performed in 300 s. All proteins were diluted in kinetics buffer (KB), a loaded sensor dipped in BLI assay buffer served as drift control. The resulting curves were aligned to the beginning of association step and a 1:1 global model was applied on the fitting.

### BLI-based binding IgG immune-complexes

A comparison of binding kinetics between IgG or IgG in complex with dimeric Fel d 1 and FcγRIIb (CD32b) was also performed using BLI assays. Briefly, SAX biosensors were saturated with 2 μg/ml of human recombinant biotinylated FcγRIIb (CD32b) followed by association with 25 nM of either monomeric IgG1 and IgG4 F127 or in pre-complexed form with 25 nM Fel d 1 for 300 s. Dissociation was also performed in 300 s in kinetics buffer. Drift control was performed with a loaded sensor dipped in kinetics buffer only. The resulting data were normalized to the highest response, using OriginPro (OriginLab Corporation, Northampton, MA, USA) software analysis.

### Statistics

Data were analyzed and statistics were performed in GraphPad Prism 9. Statistical significance is given in asterisks according to following formula: ns (not significant) for *p* > 0.05, * for *p* ≤ 0.05, ** for *p* ≤ 0.01, *** for *p* ≤ 0.001 and **** for *p* ≤ 0.0001. The specific statistical approach used is stated for every figure in the corresponding figure legend.

## Results

### IgG1 and IgG4 block activation of basophils with similar efficiency

**Figure 1:**
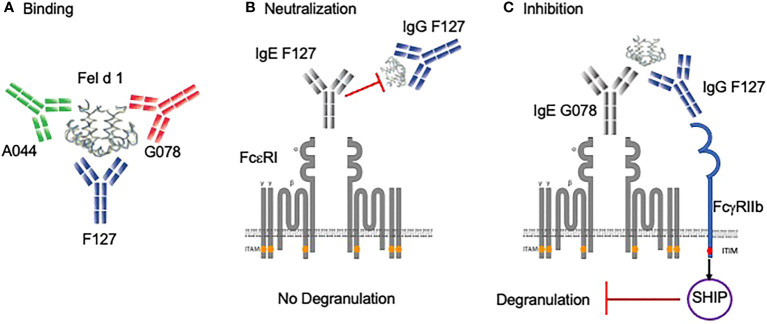
Binding and protective role of allergen-specific IgG antibodies in basophil degranulation. **(A)** Mice were immunized with the vaccine Fel d 1 coupled to Qβ-VLP. By mammalian cell display, three monoclonal antibodies (G078, A044, F127) were cloned in IgG and IgE formats that recognize non-overlapping B cell epitopes. **(B)** Anti-Fel d 1 IgG antibody (e.g. F127) having the same epitope specificity as anti-Fel d 1 IgE (e.g. F127) neutralizes Fel d 1. **(C)** Anti-Fel d 1 IgG antibody (e.g. F127) having a different specificity as anti-Fel d 1 IgE (e.g. G078) inhibit degranulation *via* FcγRIIb. Created with BioRender.com.

**Figure 2:**
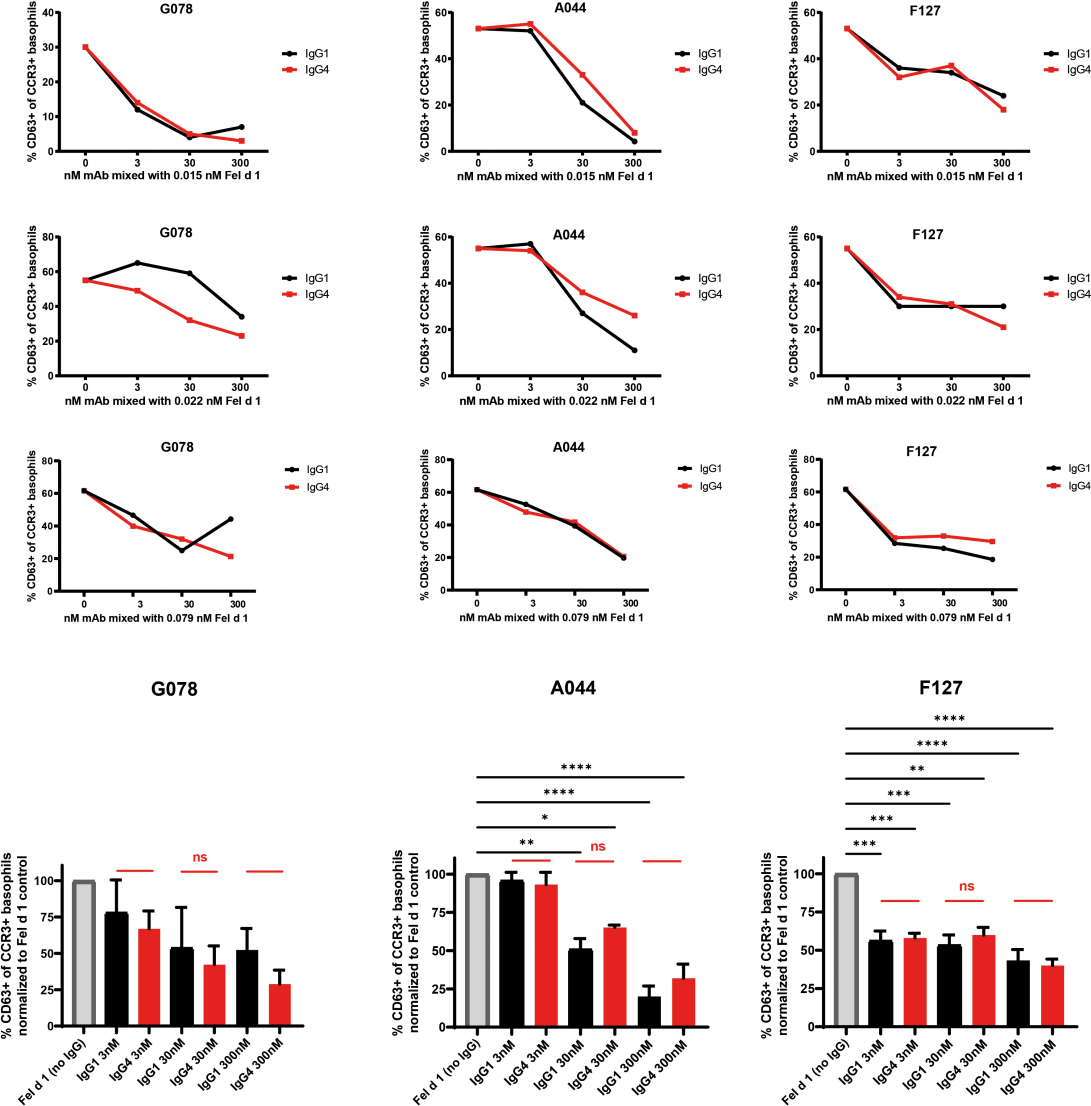
IgG1 and IgG4 mAbs equally well prevent basophil activation from cat allergic subjects. Blood from three **(A–C)** cat allergic subjects were collected and incubated with the donor specific amount of recombinant Fel d 1 (subject A: 0.015 nM; subject B: 0.022 nM; subject A: 0.079 nM) as described in material and methods. That respective amount of Fel d 1 was also pre-mixed with 3, 30, and 300 nM of Fel d 1 specific mAbs either in the format of IgG1 or IgG4. The percentage of CD63+ cells within the CCR3+ basophil population was measured. **(D)** Frequency of CD63+ cells within the CCR3+ basophil population of the three cat allergic subjects displayed in **(A–C)** normalized to frequency of CD63+ cells of the individual in the absence of IgG1/4 (maximal activation = 100%). Data are expressed as the mean of values ± SEM. For statistical analysis ordinary ANOVAs were performed. ns (not significant) for p > 0.05, * for p ≤ 0.05, ** for p ≤ 0.01, *** for p ≤ 0.001 and **** for p ≤ 0.0001.

We have previously described and characterized three monoclonal antibodies (F127, A044 and G078) against Fel d 1, the major cat allergen in humans, recognizing three non-overlapping epitopes of Fel d 1 (**Figure 1A**) (39, 40). Those variable regions were cloned in front of the human gamma 1, 4 or epsilon chain backbone to express Fel d 1 specific fully human IgG or IgE antibodies of three specificities and 2 different IgG subclasses. To test the neutralization capacity of the Fel d 1 specific IgG1 and IgG4 antibodies (**Figure 1B**), basophils from 3 different individuals (A, B and C) suffering from cat allergy were stimulated with recombinantly expressed dimeric Fel d 1 and up-regulation of CD63 was used as a read-out for basophil activation (**Figures 2A–C**). Basophils of all three individuals were activated by Fel d 1 and, most importantly, all three Fel d 1 specific mAbs (G078, A044 and F127) either of the IgG1 or IgG4 subtype were able to block basophil activation. As shown in **Figures 2A–C**, individuals differed in terms of maximal basophil activation and the kinetics of the reduction in basophil activation by the addition of antibodies. To confirm similar functionality of IgG1 and IgG4 regardless of the Fel d 1 epitope, basophil activation results were summarized by normalizing to the corresponding basophil activation of the individual without anti-Fel d 1 antibody (**Figure 2D**). We observed a dose-dependent effect of IgG inhibition which is independent of the IgG subclass for two mAbs (G078 and A044) whereas for the third one a high inhibition was already observed at the lowest concentration of IgG1 and IgG4 (**Figure 2D**). This inhibition may be a mixture of allergen-neutralization and engagement of FcγRIIb as basophils from allergic individuals likely carry polyclonal Fel d 1-specific IgE antibodies *via* FcϵRI on their surface. To dissect these 2 possibilities, we loaded basophils from non-allergic individuals with the mAbs used above but expressed as IgE.

### IgG1 and IgG4 similarly neutralize allergen and prevent primed basophils from degranulation

**Figure 3:**
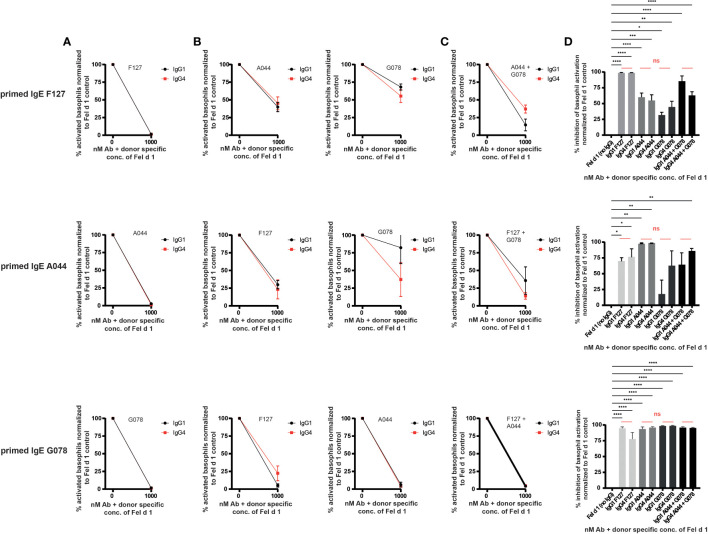
IgG1 and IgG4 prevent degranulation by neutralization and signalling *via* the inhibitory receptor FcγRIIb. Blood from three non-allergic subjects was collected and primed with Fel d 1 specific IgE. **(A)** Basophils were primed either with IgE A044, F127 or G078. Primed basophils were treated with recombinant Fel d 1 alone or pre-mixed with the same epitope expressed as IgG1 or IgG4. **(B)** Basophils were primed either with IgE A044, F127 or G078. Primed basophils were treated with recombinant Fel d 1 alone or pre-mixed with the IgG1 or IgG4 antibodies exhibiting the other two epitopes than the one used for priming. **(C)** Basophils were primed either with IgE A044, F127 or G078. Primed basophils were treated with recombinant Fel d 1 alone or pre-mixed with a combination of two IgG1 or IgG4 antibodies exhibiting the other two epitopes than the one used for priming. Donor specific Fel d 1 concentrations were evaluated for each IgE priming Ab separately as described in material and methods. Data for each combination is given as mean ± SEM. Activation of basophils of each donor was normalized to activation in the absence of IgG1/4 (=100% activation). **(D)** % inhibition of basophil activation when basophils were primed either with IgE A044, F127 or G078 and treated with Fel d 1 pre-mixed with either IgG1 or IgG4 antibodies alone or combined [as shown separately in **(A–C)**]. Data is given as mean ± SEM. Inhibition of basophil activation of each donor was normalized to inhibition in the absence of IgG1/4 (=0% inhibition). For statistical analysis ordinary ANOVAs were performed. ns (not significant) for p > 0.05, * for p ≤ 0.05, ** for p ≤ 0.01, *** for p ≤ 0.001 and **** for p ≤ 0.0001.

To test the ability of IgG1 and IgG4 to block basophil activation by neutralization (**Figure 1B**), basophils from non-allergic individuals were loaded with each of one of the IgE antibodies (G078, A044, F127) and challenged with Fel d 1 complexed with IgG antibodies of the same specificity. **Figure 3A** shows that neutralizing inhibition occurred independent of the IgG subclass, as both IgG1 and IgG4 inhibited basophil activation in a similar manner. This may also have been expected as both IgG1 and IgG4 antibodies share the same variable region for each of the 3 mAb types. Differences in neutralizing capacity therefore would have been somewhat unexpected.

### IgG1 and IgG4 prevent primed basophils from degranulation by engaging FcγRIIb

We have previously demonstrated *in vitro* and *in vivo* that the allergic reaction mediated by an anti-Fel d 1 IgE of one specificity can be blocked by a single anti-Fel d 1 IgG mAb of a different specificity (37, 40, 43). In mice, we have previously demonstrated that the mechanism of the inhibition involves the inhibitory FcγRIIb (**Figure 1C**). IgE bound on mast cells *via* the FcϵRI can bind to the allergen Fel d 1 formed as complex with IgG. The IgG antibody of the allergen complex may bind to the FcγRIIb simultaneously, which inhibits the signal cascade of the FcϵRI and prevents degranulation. To study the ability of IgG subclasses to drive this process, we performed this experiment to compare IgG1 and IgG4 for their ability to engage the FcγRIIb. Indeed, both antibody subclasses equally well impeded primed human basophils from degranulation (**Figures 3B–D**). In addition, while a single IgG-specificity distinct from the IgE used for priming, was able to block basophil activation, addition of 2 IgGs of different specificities did not significantly increase inhibition (**Figures 3C, D**). Comparison of the inhibition obtained with IgG1 and IgG4 antibody subclasses show no appreciable overall difference between the subclasses. Thus, IgG1 is at least as potent as IgG4 at blocking basophil activation.

**Figure 4:**
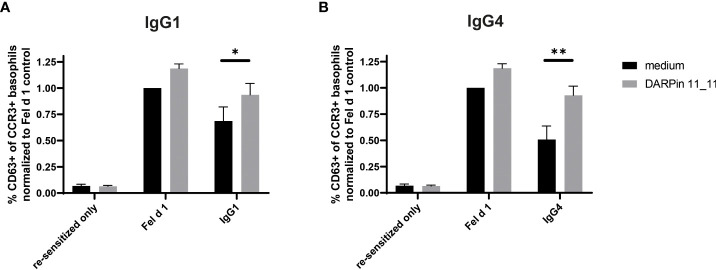
Inhibition of degranulation by IgG1 and IgG4 can be abolished through blocking of FcγRIIb. Whole blood from four non-allergic subject was collected, desensitized with a disruptive DARPin 53-79 before resensitizing with Fel d 1 specific IgE F127. Primed basophils were treated either with medium (RPMI/IL-3) or with medium containing 1000 nM DARPin 11_11 for 30 min at 37°CC. Basophils are then incubated with either Fel d 1 alone or with Fel d 1 pre-complexed with IgG1 **(A)** or IgG4 **(B)** G078 at a concentration of IgG of 100 nM). Results are expressed as the percentage of CD63 positive basophils of total basophils (CCR3+ basophils) normalized to percentage of CD63+ basophils in the Fel d 1 medium control. Data of four different donors is given as mean ± SEM. For statistical analysis two-tailed paired t-tests were performed. * for p ≤ 0.05, ** for p ≤ 0.01.

To directly demonstrate that FcγRIIb was involved in this inhibition also for human cells, we used a previously described DARPin molecule to block FcγRIIb activity (42). Prior to addition of the allergen-IgG immune complexes, basophils were incubated with the anti-FcγRIIb DARPin. Indeed, under these conditions, non-neutralizing inhibitory activity of both IgG1 and IgG4 was abrogated (**Figure 4**) and inverted the previously observed inhibition. Hence, non-neutralizing inhibition is mediated by FcγRIIb in human basophils.

### IgG1 and IgG4 bind to human FcγRIIb with similar affinity

**Figure 5:**
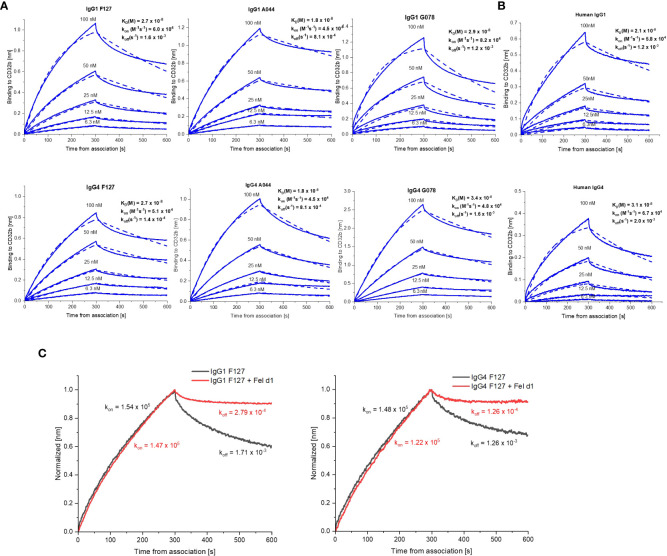
BLI sensorgrams illustrating the interaction IgG1 and IgG4 antibodies to FcγRIIb. **(A)** Binding of monomeric F127, G078 and A044 in either IgG1 or IgG4 format. **(B)** Binding of human native IgG1 or IgG4. **(C)** Binding of IgG1 and IgG4 F127 either as monomer or in pre-complexed form with Fel d 1. The on-rate (k_on_) and off-rate (k_off_) constants were compared. In both assays association and dissociation were performed 300s.

In comparison to other Fcγ receptors, the affinity of IgG antibodies to the FcγRIIb is low even if immune-complexed to antigens. We nevertheless measured the affinity of IgG1 and IgG4 antibodies to FcγRIIb (CD32b) by Biolayer Interferometry using Octet technology (44). To this end, biotinylated recombinant human FcγRIIb was immobilized on streptavidin biosensors before incubation with either F127, G078 and A044 in either IgG1 or IgG4 format. As shown in **Figure 4**, IgG1 and IgG4 antibodies bound with similar affinity to recombinant FcγRIIb (**Figure 5A**). To exclude that recombinant *in vitro* expression of the antibodies modified their binding to FcγRIIb, we repeated the experiments using IgG1 and IgG4 purified from human blood. Similar ranking of affinities was observed for both antibodies confirming previous finding with the recombinant antibodies IgG1 and IgG4 (**Figure 5B**).

We also investigated the effects of immune complex formation on the binding of IgG1 or IgG4 to FcγRIIb by BLI, performing the association step with IgG1 and IgG4 F127 either in monomeric or in complex form with dimeric Fel d 1 (**Figure 5C**). The results showed similar association rate (k_on_) for monomeric and complexed IgG1 and IgG4 whereas the dissociation rate (k_off_) was 10 times slower for the complexed forms of IgG1 and IgG4 indicating an overall higher binding for IgG immune complexes than for IgGs alone. Hence, antibody subclass may have a limited influence on FcγRIIβ binding in both monomeric and complexed form thereby confirming the results obtained in the above cellular assays.

## Discussion

AIT is the only disease modifying treatment to treat allergy. Several immune cells and mediators contribute to various degrees to the severity of the allergic reaction. Mast cells and basophils are effector cells of the early phase that are involved in tissue damage, itching and swelling. However, ablating those cells may be technically difficult and also be potentially dangerous, as they are also involved in protection against tick and helminth infections, detoxification of arthropod and reptile venoms, and, as recently shown, preserving cardiac function after myocardial infarction (45–47). Allergen-specific B cells as well as IgE and Th2 cells are therefore potentially better targets. However, allergen-specific lymphocytes do not have a unique characteristic to specifically delete them other than the T or B cell receptors which are notoriously difficult to target. AIT focusses on rendering the body more unresponsive to the allergen by changing the immune environment and induction of IgG antibodies (25, 48, 49). The high potential of IgG antibodies to reduce the severity of allergic reactions has been shown in mouse models (30, 32, 40), and more recently in a clinical study demonstrating that it is possible to treat cat allergy with monoclonal anti-Fel d 1 IgG antibodies (34). In addition, induction of allergen-specific IgG4 or allergen-specific IgE/IgG4 ratio are considered to be the best correlate of AIT efficacy. However, whether IgG4 is more potent than other IgG subclasses or merely happens to be the preferred IgG subclass induced by classical AIT is still a matter of debate. In fact, induction of IgG4 during classical AIT may actually reflect to some degree natural allergen exposure as seen in bee keepers who are not allergic to bee venom but have high serum levels of specific IgG4 (50, 51). Indeed, inducing B cell responses in the absence of innate stimuli, such as toll-like receptor ligands, may preferentially drive IgG4 responses (12, 36).

Nevertheless, the role and importance of the induced IgG subclasses during AIT may have important consequences, since use of modern vaccination regimens such as inclusion of stronger adjuvants or formulation with virus-like particles (VLPs) may favour induction of IgG1 rather than IgG4 (30). Indeed, a clinical study to treat house dust mite allergy with Der p 1 coupled to a VLP induced strong IgG1 instead of IgG4 responses (26). Another important aspect of the safety of AIT is the availability, standardization, and formulation of allergens. Given the fact that allergens coupled to VLPs do not activate basophils (31), but induce strong IgG responses compared to other approaches, may render AITs safer and more efficient in future; however, the ability of IgG1 to block the allergic reaction remains an important caveat for such new therapies.

To investigate whether IgG4 was more potent than other IgG subclasses at blocking cellular activation, we focused on basophils as primary human mast cells are very difficult to obtain. To study basophil activation in detail, we expressed 3 different monoclonal antibodies recognizing distinct epitopes on the allergen Fel d 1 in a human IgG1 and IgG4 format (40). This allowed us to study the importance of IgG subclasses in three different mechanisms of action. Specifically, we compared 1) the ability to neutralize the allergen and block basophil activation, 2) to inhibit basophil activation *via* engagement of FcγRIIb, and 3) the ability of the IgG1 and IgG4 subclasses to bind to FcγRIIb. Both IgG subclasses were important for triggering inhibitory FcγRIIb -mediated signal because blocking of the binding of both IgG1 and IgG4 to FcγRIIb with a FcγRIIb inhibitor was able to restore basophil activation. This in contrast to a previous inhibition study which showed that IgG1 has very little functional interaction with FcγRIIb whereas IgG4 had none (52). The same study demonstrated that IgG2 and IgG3 were more efficacious in interacting with FcγRIIb such that only 0.5 IgG2 per 1 antigen molecule was necessary to mediate inhibition. For comparison, in our study approximately 1000x time more IgG1 and IgG4 were used to mediate inhibition of Fel d 1 underlying the importance of the ratio of IgG:allergen to study the role of blocking antibody in the framework of inhibition experiments. In our assays we limited our comparison to IgG1 and IgG4 which are the antibody subclasses mostly involved in classical and next generation immunotherapies. We found that IgG1 and IgG4 antibodies exhibited similar efficacy at blocking basophil activation and at engaging FcγRIIb. This finding is supported by previous affinity studies of Bruhns et al. (53) which showed similar affinity for IgG1 and IgG4 using surface plasmon resonance. Using Bio-layer interferometry, we confirmed these data by showing similar affinity constants as well as on- and off-rate kinetics for binding to FcγRIIb for both IgG1 and IgG4. Moreover, we observed that the presence of the antigen makes a strong difference in the dissociation rate by making it slower. Finally, we could demonstrate that the affinity of native IgG1 for FcγRIIb is of the same order of magnitude as that of IgG4 indicating the IgG subclass has no implication for the binding to FcγRIIb ((ξ>^54^). Our data do not exclude that differences in affinity for allergens between IgG1 and IgG4 influences efficacy of AIT (55) but we formally demonstrate that the subclass per se is of minor importance. Hence, IgG4 does not have preferable characteristics for the treatment of allergy. This may indicate that induction of IgG4 is not a pre-requisite for efficient therapy but that amounts and affinities of total IgG may be more important.

## Data availability statement

The raw data supporting the conclusions of this article will be made available by the authors, without undue reservation.

## Ethics statement

The experiments involving human blood from cat-allergic and non-cat allergic patients was approved by the KEK (Zurich, Bern Switzerland) ethics committees. The patients/participants provided their written informed consent to participate in this study.

## Author contributions

MV, FT, and SZ designed, performed and interpreted experiments. GA performed experiment and corrected the manuscript. MV, FT, and MB wrote the manuscript. All authors contributed to the article and approved the submitted version.

## Funding

This project was supported by funding of the Swiss National Science Foundation (SNF grant 310030_185114 to MB).

## Acknowledgments

We thank Dr. Pascal Gasser and Prof. Alexander Eggel for the assistance in the basophil activation assay.

## Conflict of interest

MB has a financial relationship with Saiba AG involving stock ownership or payments for research activities.

The remaining authors declare that the research was conducted in the absence of any commercial or financial relationships that could be construed as a potential conflict of interest.

## Publisher’s note

All claims expressed in this article are solely those of the authors and do not necessarily represent those of their affiliated organizations, or those of the publisher, the editors and the reviewers. Any product that may be evaluated in this article, or claim that may be made by its manufacturer, is not guaranteed or endorsed by the publisher.
